# POLR2J knockdown promotes ROS-induced DDR and ferroptosis by inhibiting the STAT3-GPX4 signaling axis in LUAD

**DOI:** 10.3389/fonc.2026.1787943

**Published:** 2026-04-15

**Authors:** Jiehong Wang, Jiaojiao Chen, Xue Zhu, Yue Hao, Jing Fang, Naiyan Lu, Ke Wang, Xun Wang

**Affiliations:** 1 Department of Pulmonary and Critical Care Medicine, Affiliated Hospital of Jiangnan University, Wuxi School of Medicine, Jiangnan University, Wuxi, Jiangsu, China; 2 National Health Commission Key Laboratory of Nuclear Medicine, Jiangsu Key Laboratory of Molecular Nuclear Medicine, Jiangsu Institute of Nuclear Medicine, Wuxi, Jiangsu, China; 3 Department of Radiopharmaceuticals, School of Pharmacy, Nanjing Medical University, Nanjing, Jiangsu, China; 4 Department of Respiratory Medicine, Affiliated Jinling Hospital, Medical School of Nanjing University, Nanjing, Jiangsu, China; 5 School of Food Science and Technology, Jiangnan University, Wuxi, Jiangsu, China

**Keywords:** DDR, LUAD, POLR2J, prognosis, proliferation, reactive oxygen species, STAT3-GPX4

## Abstract

**Objective:**

Non-small cell lung cancer (NSCLC) is the malignant tumor with the highest incidence and mortality worldwide, and lung adenocarcinoma (LUAD) is currently the most common histological subtype. Although treatment strategies have advanced in recent years, the overall survival of patients with LUAD remains unsatisfactory. Therefore, it is essential to identify novel molecular targets and therapeutic strategies to improve LUAD prognosis.

**Methods:**

In this study, we analyzed online datasets to characterize POLR2J expression and its co-expressed genes in LUAD and to assess their prognostic value. LUAD cell lines with POLR2J knockdown or overexpression were established. The biological functions of POLR2J in LUAD were evaluated using MTT assays, colony formation assays, and reactive oxygen species (ROS) assays. In addition, the effect of POLR2J on the STAT3-GPX4 signaling pathway in LUAD cells was investigated by transcriptomic analysis and Western blotting.

**Results:**

POLR2J expression was significantly upregulated in LUAD cells, and high POLR2J expression was associated with poor prognosis in LUAD patients. *In vitro*, POLR2J overexpression markedly enhanced LUAD cell proliferation and organoid model growth, while reducing ROS production and cell death. Conversely, POLR2J knockdown significantly suppressed cell proliferation and increased ROS-induced DNA damage response (DDR)c. Furthermore, POLR2J knockdown led to a marked decrease in the expression of proteins involved in the STAT3-GPX4 pathway and promoted ROS production, whereas IL-6 treatment effectively reversed the POLR2J knockdown–mediated inhibition of the p-STAT3/STAT3 pathway and reduced ROS levels.

**Conclusions:**

Our findings indicate that POLR2J functions as an oncogene in LUAD, is associated with poor patient prognosis, and may promote LUAD cell proliferation by activating the STAT3-GPX4 signaling pathway. These results suggest that POLR2J represents a potential therapeutic target for LUAD and may provide a promising strategy for targeted anti-LUAD therapy.

## 1 Introduction

Lung cancer is the most commonly diagnosed malignancy worldwide, with adenocarcinoma being its predominant histological subtype (1). For patients with early-stage disease, treatment strategies such as surgery combined with adjuvant radiotherapy, chemotherapy, or targeted therapy have markedly improved survival outcomes (2). However, for those diagnosed at advanced stages, even the presence of actionable driver mutations does not guarantee favorable prognosis, as a considerable proportion of patients continue to experience poor survival (3). Tumor heterogeneity and the inevitable development of drug resistance remain major challenges that hinder therapeutic efficacy (4). Therefore, identifying novel molecular targets for LUAD is of great importance. Moreover, elucidating the underlying mechanisms is essential for enhancing diagnostic precision and optimizing therapeutic strategies.

Accumulating evidence indicates that dysregulation of the transcriptional machinery plays a pivotal role in tumor initiation, progression, and therapeutic resistance (5). POLR2J, a core subunit of RNA polymerase II, is involved in transcription initiation, elongation, and mRNA processing (6). Evidence linking POLR2J to cancer was first reported in studies of lung tumor expression profiling. In a cohort of 50 lung tumor samples, POLR2J-along with eight other genes-was found to be upregulated by approximately twofold compared with normal bronchial epithelial cells (7). In basal-like breast cancer, POLR2J has been classified as a potential oncogene, suggesting that it may possess broad oncogenic potential across different tumor types (8). Although previous studies have suggested that POLR2J may contribute to the proliferation and survival of various cancer types (9), its biological functions and regulatory mechanisms in LUAD remain largely unclear. Preliminary bioinformatic analyses have shown that POLR2J may be aberrantly activated in several malignancies and associated with unfavorable clinical outcomes (10); however, functional and mechanistic evidence in LUAD is still limited.

Given its role in transcriptional regulation, POLR2J may co-opt critical signaling networks that sustain malignant growth and enhance invasive potential. Among these, STAT3-a transcription factor recurrently deregulated across human cancers-emerges as a compelling candidate (11). STAT3 is rapidly activated by cytokine- and growth factor-driven phosphorylation, enabling its nuclear translocation and the induction of gene programs governing proliferation, stemness, immune evasion, and survival (12). STAT3 orchestrates key programs in energy metabolism, redox homeostasis and cancer progression, positioning it as a central node in the regulatory circuitry of tumor cells (13). Persistent STAT3 activation is a defining feature of tumor progression and poor prognosis, including in lung cancer (12). Its integral function in redox control and tumor cell viability positions STAT3 as a plausible downstream effector through which POLR2J may exert oncogenic influence. Defining the crosstalk between POLR2J and STAT3 may therefore reveal previously unrecognized mechanisms driving LUAD pathogenesis.

Despite emerging evidence that POLR2J may contribute to tumorigenesis, its functional significance and mechanistic involvement in LUAD have remained poorly defined. In particular, whether POLR2J intersects with key oncogenic signaling pathways or participates in redox regulation-processes central to LUAD progression-has not been previously explored. The lack of such mechanistic insight has limited our understanding of how POLR2J may drive malignant behavior. In this context, we identify POLR2J as a previously unrecognized driver of LUAD progression and delineate a POLR2J-STAT3-GPX4-ROS signaling axis at the core of its oncogenic activity. POLR2J regulates intracellular oxidative stress by modulating the STAT3-GPX4 signaling axis, thereby shaping transcriptional programs that sustain tumor growth, survival and metastatic competence. These findings close a critical knowledge gap by linking POLR2J to STAT3-GPX4-mediated redox regulation and position POLR2J as a promising biomarker and actionable vulnerability in LUAD.

## 2 Materials and methods

### 2.1 Chemicals and reagents

Napabucasin and IL-6 were purchased from MedChemExpress (Shanghai, China). Primary antibodies used in this study included: γ-H2AX (ab81299), p-STAT3 (Y705) (ab267373), and GAPDH (ab8245), all obtained from Abcam (Cambridge, MA, USA). STAT3 (60199-1-Ig) and GPX4(67763-1-1g) were sourced from Proteintech (Wuhan, China).

### 2.2 Cell lines and culture

The human lung squamous carcinoma cell line H226 was obtained from ATCC (American Type Culture Collection, VA, USA). The human lung squamous carcinoma cell line H1781 and the human bronchial epithelial cell line BEAS-2B were obtained from the National Collection of Authenticated Cell Cultures (Shanghai, China). Cells were cultured in RPMI-1640 medium supplemented with 10% (v/v) fetal bovine serum (FBS) and 1% penicillin/streptomycin (P/S) in a humidified atmosphere containing 5% CO_2_ at 37 °C.

### 2.3 Cell viability assays

Cells were seeded into 96-well plates at a density of 1 × 10_4_ cells/well and treated with Napabucasin. After treatment, 5 mg/mL MTT (ST316, Beyotime) was added to each well and incubated for 4 h. Absorbance was measured at 450 nm using a microplate reader (SpectraMax M5, Molecular Devices, CA, USA). Cells were seeded into 6-well plates at 1,000 cells/well and cultured, with medium replaced every 3 days. At the endpoint, cells were fixed with methanol for 15 min, stained with 1% crystal violet, and colonies were counted.

### 2.4 3D Cell culture

For 3D culture, round-bottom 96-well plates were coated with 40 μL of coating solution (C0366S, Beyotime) overnight at 37 °C. H1781 and H226 cells (passage 5–10) were seeded at 5 × 10³ cells per well and centrifuged at 300 × g for 5 min to promote aggregation. After 48 h of incubation, spheroid formation was observed and imaged. Spheroid volume was calculated using ImageJ software based on diameter measurements. At least six spheroids per group were analyzed in triplicate experiments.

### 2.5 Wound healing assay

Cells were seeded in 6-well plates and cultured to confluence. A scratch was made with a 200 μL pipette tip, and detached cells were removed by PBS washing. Cells were then cultured in low-serum medium, and wound closure was photographed at indicated time points. The wound area was quantified using ImageJ software, and migration rates were calculated. Experiments were performed in triplicate.

### 2.6 Transwell invasion assay

Cell invasion was evaluated using Transwell chambers coated with Matrigel. Briefly, 5 × 10_4_ cells in serum-free medium were seeded into the upper chamber, and medium containing 10% FBS was added to the lower chamber. After 24 h of incubation, non-invading cells were removed, and invading cells were fixed, stained with crystal violet, and counted under a microscope. Experiments were performed in triplicate.

### 2.7 Cell apoptosis assay

Cells were seeded into 24-well plates at 5 × 10_4_ cells/well. Cells were fixed with 4% paraformaldehyde for 30 min, permeabilized for 7 min at room temperature with enhanced immunostaining permeabilization buffer (P0097, Beyotime), and incubated with Tunel reaction solution (C1090, Beyotime) for 60 min at 37°C. Nuclei were counterstained with DAPI, and fluorescence images were acquired using an Olympus IX53 fluorescence microscope.

### 2.8 Western blot analysis

Cells were lysed in RIPA buffer, and protein concentrations were determined using the BCA Protein Assay Kit (Beyotime). Equal amounts of protein were separated by 15% SDS-PAGE and transferred to PVDF membranes. Membranes were blocked, incubated with primary antibodies at 4 °C overnight, followed by HRP-conjugated secondary antibodies at 37 °C for 2 h. Signals were visualized using an ECL detection kit (Beyotime).

### 2.9 Intracellular ROS analysis

Intracellular ROS levels were measured using the DCFH-DA Cellular ROS Detection Kit (Cat. No. C1060, Beyotime, Shanghai, China) according to the manufacturer’s instructions, with minor modifications. Cells were seeded in 24-well plates at a density of 5×10 (4) cells per well and cultured overnight. After treatment with the indicated agents, cells were incubated with 10 µM DCFH-DA at 37 °C for 20 min in the dark. Cells were then washed three times with PBS to remove excess probe. Fluorescence images were captured using an Olympus IX53 inverted fluorescence microscope (Olympus, Japan) under identical exposure settings. Fluorescence intensity per cell was quantified using ImageJ software (NIH, USA), and results were normalized to untreated control cells. Hydrogen peroxide (100 µM, 30 min) was used as a positive control.

### 2.10 EdU assay

Cell proliferation was evaluated using the BeyoClick™ EdU Cell Proliferation Kit with Alexa Fluor 488 (Cat. No. C0071S, Beyotime, Shanghai, China) according to the manufacturer’s instructions, with minor modifications. Briefly, cells were seeded in 24-well plates at a density of 5×10 (4) cells per well and allowed to adhere overnight. Following treatment with the indicated agents, cells were incubated with 10 µM 5-ethynyl-2’-deoxyuridine (EdU) for 2h at 37 °C to label newly synthesized DNA. Cells were then washed twice with PBS, fixed with 4% paraformaldehyde for 15 min at room temperature, and permeabilized with 0.5% Triton X-100 for 20 min. EdU incorporation was detected by reaction with the Alexa Fluor 488 azide dye according to the kit protocol. Nuclei were counterstained with Hoechst 33342 (10 µg/mL) for 10 min. Fluorescence images were captured using an Olympus IX53 inverted fluorescence microscope (Olympus, Japan) under consistent exposure settings. The proliferation rate was calculated as the percentage of EdU-positive cells relative to the total number of Hoechst-stained nuclei. At least five randomly selected fields per well were analyzed using ImageJ software (NIH, USA).

### 2.11 Quantitative real-time PCR

Total RNA was extracted using Trizol reagent (Cat. No. R0011, Beyotime, Shanghai, China) according to the manufacturer’s protocol. RNA (1 µg) was reverse transcribed into cDNA using the PrimeScript™ RT-PCR kit (Cat. No. R211, Vazyme, Nanjing, China). qRT-PCR was performed on an ABI 7500 Fast Real-Time PCR System (Thermo Fisher, USA) using SYBR Premix Ex Taq™ (Cat. No. Q712, Vazyme). Primers were designed to specifically amplify target genes, with GAPDH serving as the internal control. Relative gene expression was calculated using the 2^−^ΔΔCt method. All reactions were conducted in triplicate, and data are presented as mean ± SD. Statistical analysis was performed using one-way ANOVA with Tukey’s *post hoc* test, and p < 0.05 was considered statistically significant. The primer sequences used were as follows: POLR2J: Forward: 5′-AGGGCGAGAAGAAACAACTCC-3′, Reverse: 5′-TCGGTGATGGCGTTGGTAAA-3′; GAPDH: Forward: 5′-GGAGCGAGATCCCTCCAAAAT-3′, Reverse: 5′-GGCTGTTGTCATACTTCTCATGG-3′.

### 2.12 Cell transfection

For gene knockdown or overexpression, cells were transfected with specific siRNAs targeting POLR2J, or with pcDNA3.1-POLR2J overexpression plasmid (GenePharma, Shanghai, China) using Lipofectamine 3000 (Life Technologies, USA) following the manufacturer’s instructions. Briefly, cells were seeded to reach ~70% confluence at the time of transfection. Forty-eight hours post-transfection, knockdown or overexpression efficiency was verified by Western blotting before subsequent experiments.

### 2.13 Chromatin immunoprecipitation-qPCR

Chromatin immunoprecipitation (ChIP) assays were performed using a commercial Chromatin Immunoprecipitation Kit (Cat. No. P2078, Beyotime, Shanghai, China) with minor modifications. Briefly, cells were cultured to ~80% confluence and cross-linked with 1% formaldehyde for 10 min at room temperature. The cross-linking reaction was quenched with 0.125 M glycine for 5 min, followed by two washes with ice-cold PBS. Cells were harvested, lysed to isolate nuclei, and chromatin was sheared by sonication using a Bioruptor sonicator (Diagenode, Belgium) to obtain DNA fragments averaging 200–500 bp. Sheared chromatin was incubated overnight at 4 °C with either 5 µg anti-STAT3 antibody (specify catalog number and supplier) or normal rabbit IgG as a negative control, both pre-bound to protein A/G magnetic beads. Immunoprecipitates were washed extensively, eluted, and reverse cross-linked at 65 °C for 4 h in the presence of proteinase K. DNA was purified using the kit’s spin columns. Quantitative PCR (qPCR) was conducted using SYBR Green Master Mix (specify brand) on a QuantStudio™ 6 Flex Real-Time PCR System (Applied Biosystems, USA). Primers targeting the putative STAT3-binding site within the GPX4 promoter were used. Enrichment of STAT3 binding was calculated as a percentage of input chromatin and normalized to IgG controls.

### 2.14 Intracellular iron assay

Intracellular iron levels were quantified using a commercial iron assay kit (MAK025, Sigma-Aldrich, Munich, Germany). Following treatment, cells were collected and lysed in Iron Assay buffer, followed by homogenization and centrifugation at 16,000×g for 10 min at 4 °C. The resulting supernatant was incubated with an iron probe for 30 min at room temperature. Absorbance was then measured at 593 nm using a microplate fluorometer (Molecular Devices).

### 2.15 ELISA

Human IL-6 was measured using an enzyme-linked immunosorbent assay (ELISA) kit (Solarbio, China; catalog number: SEKH-0013) according to the manufacturer’s instructions. Briefly, cell culture supernatants were collected and centrifuged at 1000 × g for 10 min at 4 °C to remove debris. All reagents and samples were brought to room temperature for 30 min before use.

### 2.16 Co-immunoprecipitation assay

For co-immunoprecipitation, cells were lysed in IP lysis buffer (P0013, Beyotime, Shanghai, China) supplemented with protease and phosphatase inhibitors. Protein concentrations were determined using the BCA protein assay. One milligram of lysate was incubated with protein A+G agarose (P2055, Beyotime, Shanghai, China) and the corresponding specific antibody at 4 °C for 4 hours. After centrifugation, the beads were washed three times with 1× TBS buffer (ST665, Beyotime, Shanghai, China), and bound proteins were eluted with 2× SDS-PAGE sample loading buffer. The resulting samples were then analyzed by Western blot as described above.

### 2.17 Animal experiments

All the animal experiments were approved by the Animal Experimental Ethics Committee of Jiangsu Institute of Nuclear Medicine (Wuxi, China). Four-week-old female BALB/c nude mice were obtained from Weitong Lihua Laboratory Animal Technology Co. LTD (Zhejiang, China) and housed in a pathogen-free environment. The mice were randomly divided into three groups (n=5):NC-Vector, POLR2J(KO), and POLR2J(OE). Different cells (2 × 10^6^ each) were subcutaneously injected into the right shoulder of each mouse. Tumor size was measured every three days, and the volume of each tumor was calculated by the formula of 1/2×(length×width (2)). After the 18-day observation period, the mice were euthanized and the tumor tissues were collected, weighed, and analyzed using immunohistochemistry.

### 2.18 Immunohistochemistry

Tumor tissues were embedded in paraffin and sectioned into 4 μm-thick slices. Hematoxylin and eosin (H&E) staining and Ki67 immunohistochemical staining were performed to assess tissue morphology and proliferation, respectively. The expression of POLR2J was evaluated via immunohistochemical staining using a DAB kit for visualization. Images were recorded with a light microscope (Olympus IX53).

### 2.19 MicroPET imaging

For radiolabeling, 50 μg of NOTA-PK10 (targeting integrin αVβ3), 6 μL of 2 mM AlCl_3_, and 50 μL of target water containing ^18^F (~100 MBq) were mixed in a sodium acetate-acetonitrile buffer and incubated at 95°C for 10 min. The mixture was purified using a C18 column, and the labeled compound [^18^F]F-NOTA-PRGD_2_ was eluted with 10 mM HCl in ethanol (0.3 mL). Approximately 3.7 MBq of the labeled compound was administered to mice via tail vein injection under isoflurane anesthesia. After 1 h, dynamic imaging was performed using an Inveon microPET scanner (Siemens Medical Solutions, Erlangen, Germany). Radioactivity in the tissues was quantified using a Perkin-Elmer γ-counter (Waltham, MA, USA). Data were presented as the percentage of the injected dose per gram of tissue (%ID/g).

### 2.20 Statistical analysis

Data are presented as mean ± SD from at least three independent experiments. Comparisons between two groups were performed using Student’s t-test, while multiple-group comparisons were analyzed by one-way ANOVA followed by Tukey’s *post hoc* test (GraphPad Prism 8.0). A p-value < 0.05 was considered statistically significant.

## 3 Results

### 3.1 POLR2J was overexpressed and predicted poor prognosis in LUAD patients

**Figure 1:**
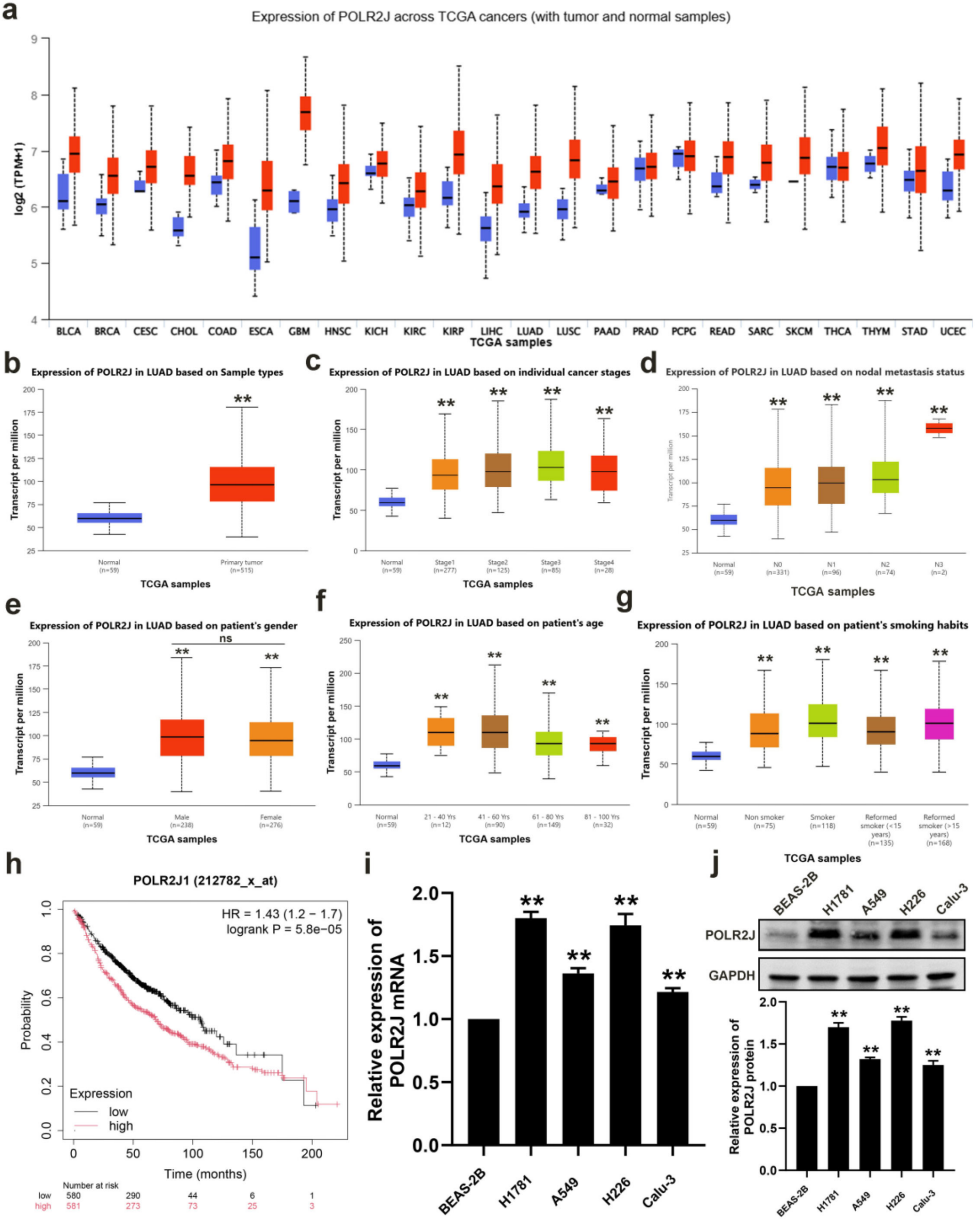
POLR2J was overexpressed and predicted poor prognosis in LUAD patients. **(a)** The expression of POLR2J across multiple cancer types (pan-cancer) was detected and analyzed using the UALCAN database. **(b)** High expression of POLR2J in LUAD. **(c–g)** Correlation between POLR2J mRNA expression levels and different clinical characteristics. **(h)** Kaplan-Meier analysis of the correlation between POLR2J expression and overall survival (OS) in LUAD. **(i)** RT-qPCR detection of POLR2J mRNA expression levels in LUAD cell lines. **(j)** Western blot detection of POLR2J protein expression levels in LUAD cell lines. The results were presented as the Means ± standard deviation (SD), with n=3. *p < 0.05, **p < 0.01 vs. the control group.

Initially, through the search and analysis of POLR2J mRNA in the UALCAN database, we found that POLR2J mRNA levels were significantly upregulated in LUAD tissues compared with normal tissues (**Figures 1a, b**). Further analysis of the correlation between POLR2J mRNA expression level and different clinical characteristics using UALCAN database data indicated that higher POLR2J mRNA expression was significantly associated with advanced individual tumor stage and lymph node metastasis (**Figures 1c, d**). Subgroup analysis based on gender, age, and smoking habit demonstrated that POLR2J mRNA expression was consistently upregulated in tumor tissues than in normal tissues in all subgroups (**Figures 1e–g**). In addition, the Kaplan-Meier plot of POLR2J from the TCGA-LUAD dataset obtained from the Kaplan-Meier Plotter also predicted poor prognosis in LUAD patients with high POLR2J expression (**Figure 1h**). Therefore, we further detected the mRNA and protein levels of POLR2J in the human normal lung epithelial cell line BEAS-2B and LUAD cell lines (Calu-3, H1781, H266, A549). As shown in **Figures 1i, j**, the expression levels of POLR2J mRNA and protein in LUAD cell lines were significantly higher than those in BEAS-2B cells. The present study demonstrated that POLR2J is significantly overexpressed in LUAD, and the overexpression of POLR2J is associated with poor prognosis in LUAD patients, suggesting that it may play a crucial regulatory role in the pathogenesis and progression of LUAD.

### 3.2 POLR2J knockdown inhibited cell proliferation and organoid model growth in LUAD cells

**Figure 2:**
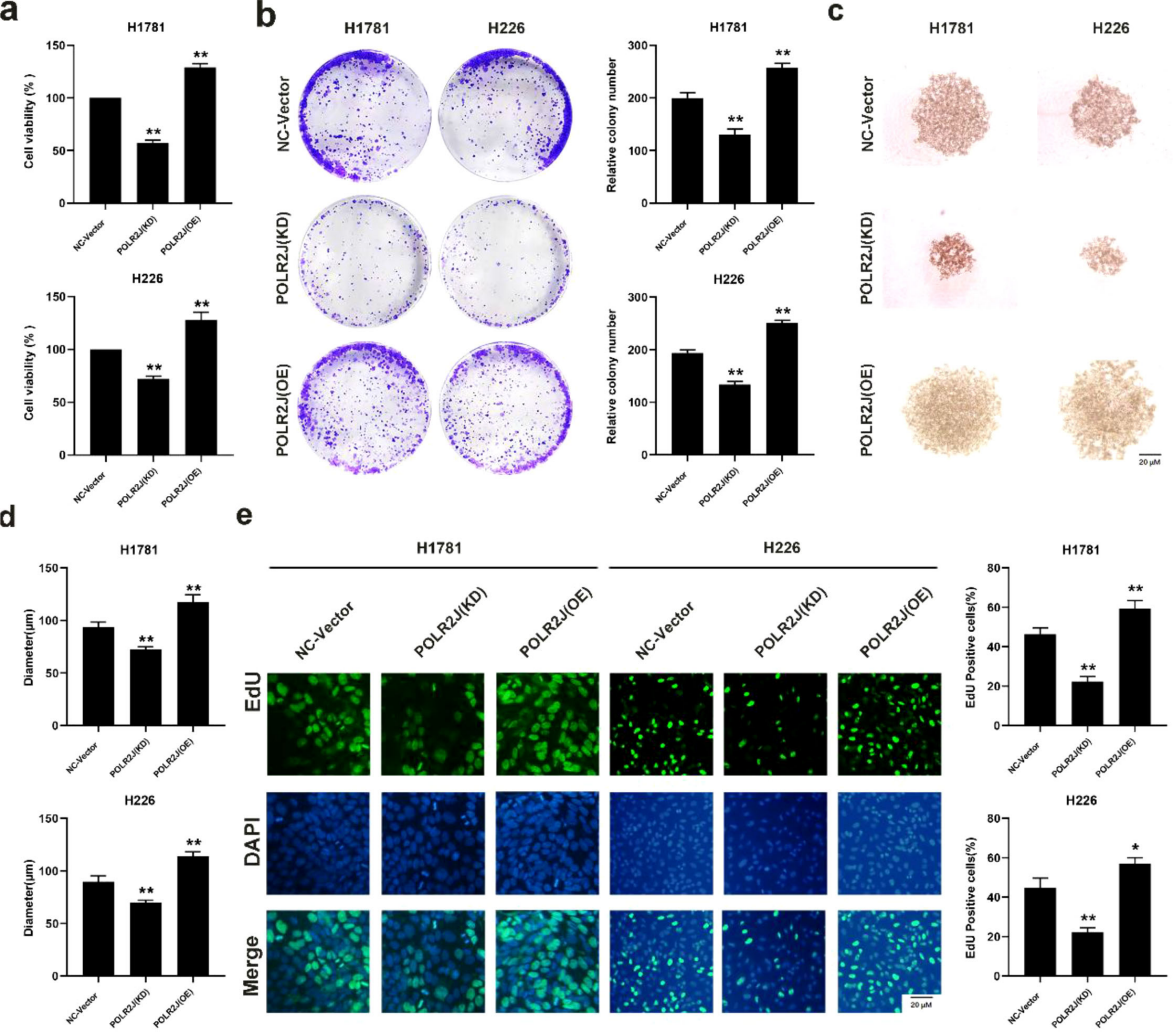
POLR2J knockdown inhibited cell proliferation and organoid model growth in LUAD cells. **(a)** MTT detection of the effect of POLR2J knockdown or overexpression on the proliferation of the LUAD cell lines H1781 and H226. **(b)** Effect of POLR2J knockdown or overexpression on the proliferative capacity of LUAD cell lines H1781 and H226 as determined by clonogenic assay. **(c, d)** Effect of POLR2J knockdown or overexpression on proliferative capacity of LUAD organoid models derived from H1781 and H226 cells. **(e)** Effect of POLR2J knockdown or overexpression on the proliferation of the LUAD cell lines H1781 and H226, as detected by EdU staining assay. The results were presented as the Means ± standard deviation (SD), with n=3. *p < 0.05, **p < 0.01 vs. the control group.

Building on the aforementioned findings, the present study further investigated the biological function of POLR2J in LUAD cells. Cell viability and proliferation were evaluated using MTT and colony formation assays. The results demonstrated that knockdown of POLR2J significantly inhibited the cell viability of LUAD cell lines H1781 and H226, while overexpression of POLR2J remarkably promoted these effects (**Figures 2a, b**). A subsequent EdU staining assay confirmed the reduction in cell proliferation in POLR2J-knockdown cells (**Figure 2e**). In addition, under three-dimensional culture conditions, knockdown of POLR2J significantly suppressed the growth of LUAD organoid models, whereas overexpression of POLR2J notably enhanced the growth of LUAD organoid models (**Figures 2c, d**). Collectively, these findings suggest that the expression level of POLR2J exerts a substantial regulatory role in the proliferation of LUAD cells, and inhibition of POLR2J expression can effectively suppress LUAD cell proliferation.

### 3.3 POLR2J knockdown inhibited LUAD cells migration and invasion

**Figure 3:**
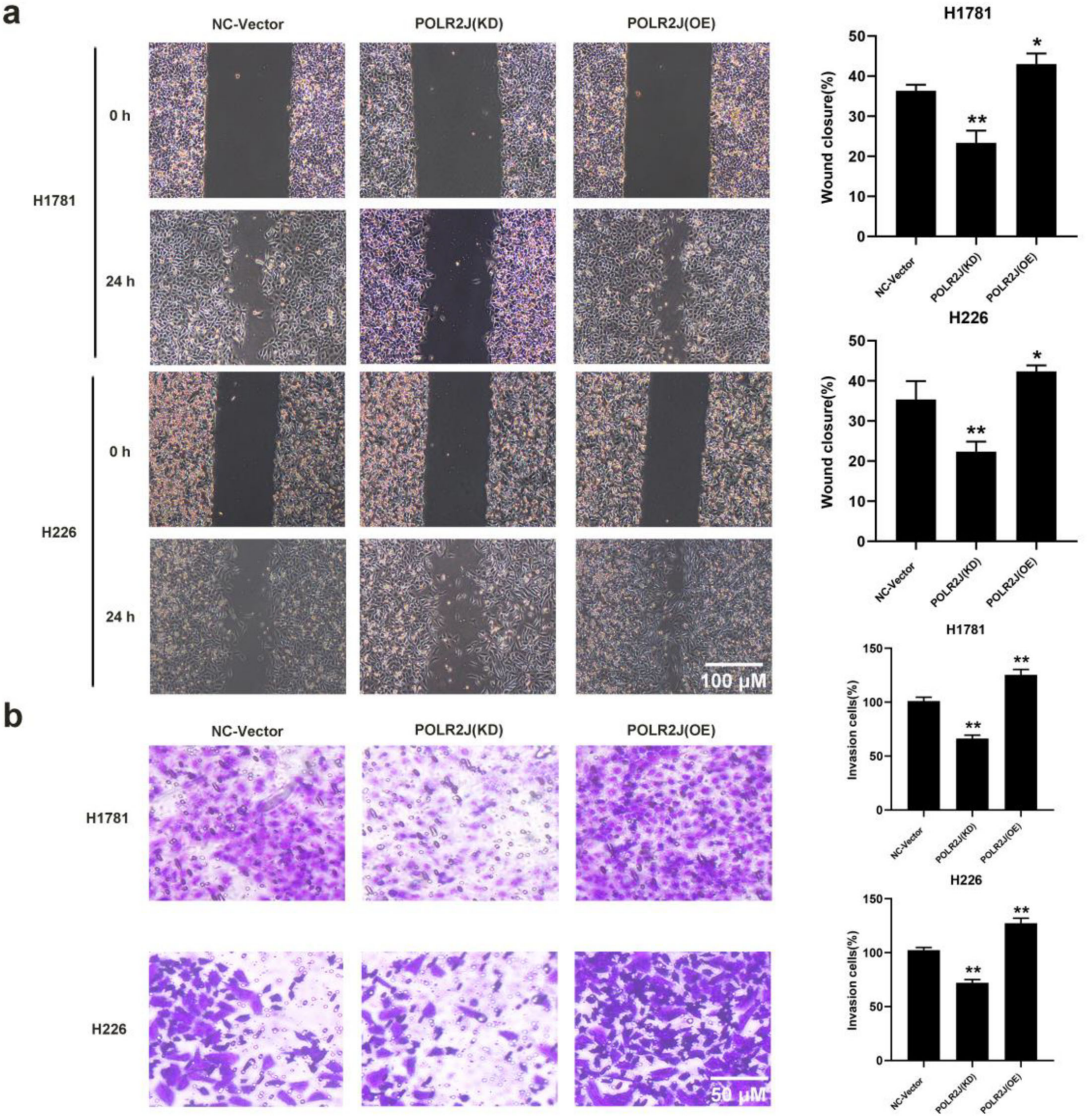
POLR2J knockdown inhibited LUAD cells migration and invasion. **(a)** Representative images of wound healing assays and quantitative analysis of migration distance in NSCLC cells. **(b)** Representative images of Transwell invasion assays and quantitative analysis of invaded cell numbers in NSCLC cells. The results were presented as the Means ± standard deviation (SD), with n=3. *p < 0.05, **p < 0.01 vs. the control group.

Epithelial-Mesenchymal Transition (EMT) is considered a key process in initiating tumor invasion and metastasis, affecting cell shape, adhesion, and motility (14). In this study, wound healing assays were performed to examine the effect of POLR2J expression on the migration ability of LUAD cells. The results showed that compared with the control group, POLR2J knockdown significantly inhibited the migration of H1781 and H226 cells, whereas POLR2J overexpression markedly induced the migration of both cell lines (**Figure 3a**). Furthermore, Transwell assays were conducted to evaluate the effect of POLR2J expression on LUAD cell invasion. The results demonstrated that compared with the control group, POLR2J knockdown significantly suppressed the invasion of H1781 and H226 cells, while POLR2J overexpression notably enhanced the invasion of both cell lines (**Figure 3b**). These findings suggest that inhibition of POLR2J expression effectively suppresses the migration and invasion of LUAD cells.

### 3.4 POLR2J knockdown triggers DDR and cell death in a ROS-dependent manner in LUAD cells

**Figure 4:**
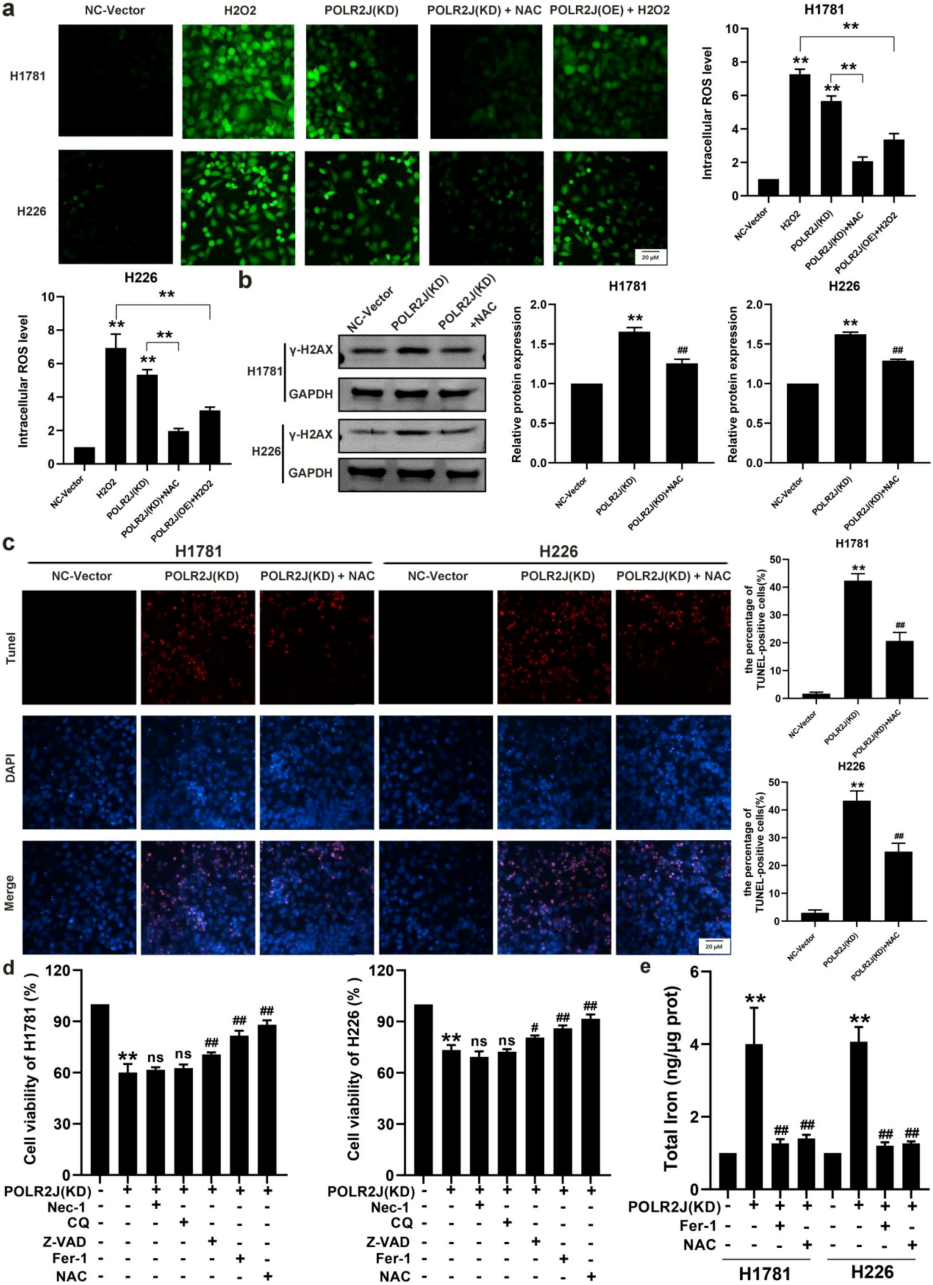
POLR2J knockdown triggers DDR and cell death in a ROS-dependent manner in LUAD cells. **(a)** DCFH-DA probe detection of the effect of POLR2J knockdown or overexpression on reactive oxygen species (ROS) production in LUAD cell lines H1781 and H226. **(b)** Western blot detection of γ-H2AX protein expression levels in LUAD cell lines H1781 and H226. **(c)** Effect of POLR2J knockdown or overexpression on the DNA damage of the LUAD cell lines H1781 and H226, as detected by Tunel staining assay. **(d)** Effects of the necroptosis inhibitor Nec-1, chloroquine (CQ), the pan-caspase inhibitor Z-VAD, the ferroptosis-specific inhibitor ferrostatin-1 (Fer-1), and the ROS scavenger NAC on the viability of POLR2J knockdown cells. **(e)** Effects of Fer-1 and NAC on intracellular ferrous iron levels in POLR2J knockdown cells. The results were presented as the Means ± standard deviation (SD), with n=3. *p < 0.05, **p < 0.01 vs. the control group; #*p* < 0.05, ##*p* < 0.01 vs. the POLR2J(KD).

Oncogene activation elicits an increase in intracellular reactive oxygen species (ROS) accumulation, thereby provoking DNA replication stress and culminating in cell death (15), DCFH-DA staining was used to measure intracellular reactive oxygen species (ROS) production. As expected, POLR2J knockdown significantly promoted ROS generation, indicated by increased green fluorescence, which could be reversed by treatment with the ROS scavenger N-acetylcysteine (NAC). Hydrogen peroxide (H_2_O_2_) is a universal inducer of cellular ROS, and POLR2J overexpression notably reduced ROS production in H1781 and A549 cells following H_2_O_2_ treatment (**Figure 4a**). In addition, POLR2J knockdown significantly upregulated the expression of γ-H2AX protein, a marker of DDR (DNA-damage response). After ROS scavenging by NAC and GSH, the expression of γ-H2AX protein was significantly decreased, indicating that most of the aforementioned DNA damage was repaired (**Figure 4b**, **Supplementary Figure 1**). To verify the association between excessive ROS, oxidative DNA damage, and death in LUAD cells, we investigated the effect of NAC on cell death induced by POLR2J knockdown. The results showed that NAC significantly inhibited the proportion of dead cells in POLR2J-knockdown H1781 and H226 cells (**Figure 4c**). Inhibitor screening revealed that only Fer-1 and NAC significantly reversed the cell death caused by POLR2J knockdown. Consistently, POLR2J knockdown markedly increased intracellular iron levels (**Figures 4d, e**), indicating that POLR2J deficiency induces ferroptosis in LUAD cells. Collectively, the findings of this study suggest that inhibition of POLR2J expression can effectively promote ROS-dependent DNA-damage response and ferroptosis in LUAD cells.

### 3.5 POLR2J knockdown regulates ROS production by inhibiting the STAT3 signaling pathway in LUAD cells

**Figure 5:**
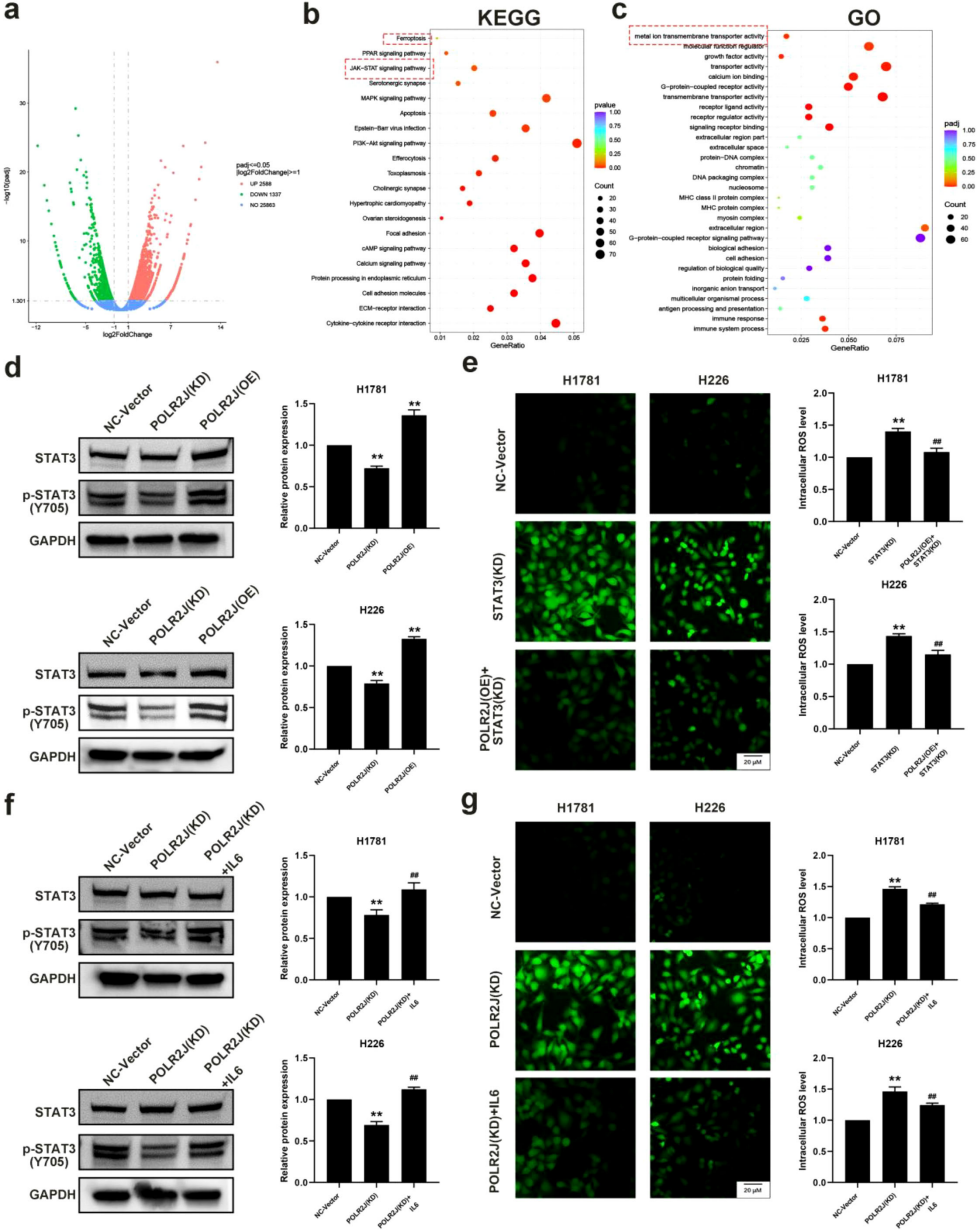
POLR2J knockdown regulates ROS production in LUAD cells by inhibiting the STAT3 signaling pathway. **(a)** Volcano plots of DEGs with log2 fold change ≥ 1 (p-value ≤ 0.05). **(b)** Kyoto Encyclopedia of Genes and Genomes analysis of DEGs. **(c)** Gene ontology analysis of DEGs. **(d, f)** Western blot detection of p-STAT3/STAT3 protein expression levels in LUAD cell lines H1781 and H226. **(e, g)** Detection of reactive oxygen species (ROS) content in LUAD cell lines H1781 and H226 using DCFH-DA probe. The results were presented as the Means ± standard deviation (SD), with n=3. *p < 0.05, **p < 0.01 vs. the control group; #*p* < 0.05, ##*p* < 0.01 vs. the siSTAT3 or POLR2J (KD).

Transcriptome sequencing was performed on LUAD cells from the control group and the POLR2J knockdown group, revealing a total of 3925 differentially expressed genes (DEGs) between the two groups, among which 2588 genes were significantly upregulated and 1337 genes were significantly downregulated (**Figure 5a**). KEGG pathway enrichment analysis of DEGs demonstrated that pathways such as the PPAR signaling pathway, JAK-STAT signaling pathway, MAPK signaling pathway, PI3K-AKT signaling pathway, and cAMP signaling pathway were significantly enriched (**Figure 5b**). Additionally, GO enrichment analysis of DEGs showed significant enrichment in biological processes including redox imbalance, signal receptor binding, DNA damage repair, and cell death (**Figure 5c**). Consistent with the findings of our study, POLR2J knockdown can promote the production of ROS and DDR in LUAD cells. Furthermore, these results suggest that POLR2J knockdown may facilitate the death of LUAD cells by regulating the STAT3 signaling axis.

Subsequently, to elucidate the potential mechanism of POLR2J in LUAD, Western blot results demonstrated that silencing of POLR2J significantly reduced the phosphorylation level of STAT3, while overexpression of POLR2J increased the phosphorylation level of STAT3 (**Figure 5d**). A large body of evidence has shown that STAT3 plays a pivotal role in tumor-derived ROS production. To further clarify whether POLR2J regulates ROS levels through the STAT3 signaling pathway, we investigated whether overexpression of POLR2J could rescue the STAT3 silencing-mediated ROS production in LUAD cells. DCFH-DA staining assay indicated that STAT3 knockdown significantly elevated intracellular ROS levels in LUAD cells, whereas overexpression of POLR2J notably attenuated intracellular ROS levels in STAT3-knockdown LUAD cells (**Figure 5e**). Further experiments in POLR2J-silenced cells revealed that POLR2J knockdown or overexpression did not affect IL6 expression levels (**Supplementary Figure 2**). However, the addition of exogenous IL6 reduced ROS levels in POLR2J-silenced LUAD cells by enhancing STAT3 phosphorylation (**Figures 5f, g**). Collectively, these results suggest that POLR2J can participate in the regulation of intracellular ROS levels and STAT3 phosphorylation in LUAD cells by activating STAT3.

### 3.6 POLR2J promotes GPX4 expression through interaction with STAT3

**Figure 6:**
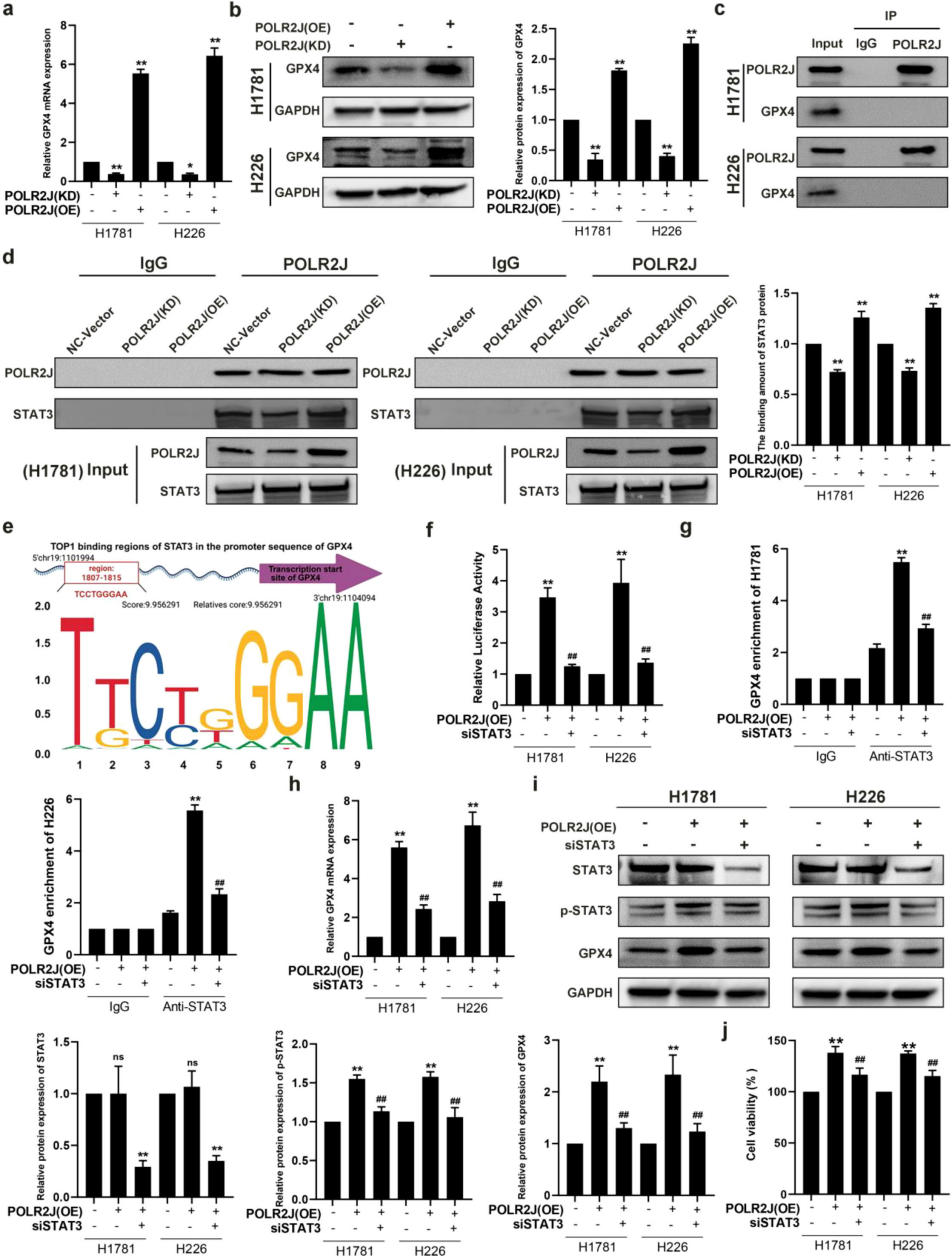
POLR2J promotes GPX4 expression through interaction with STAT3. **(a, b)** GPX4 mRNA and protein expression levels in POLR2J knockdown or overexpression cells. **(c, d)** The interaction between POLR2J and GPX4 or STAT3 was examined by co-immunoprecipitation (Co-IP) assays. **(e)** Predicted GPX4 binding motifs and sequences from the JASPAR website. **(f)** STAT3 binding to GPX4 promoter was evaluated by dual luciferase reporter assay. **(g)** Chromatin immunoprecipitation with anti-STAT3 antibody-coupled qPCR assays demonstrates the binding of STAT3 to the GPX4 promoter region. **(h, i)**: The expression of GPX4 was detected by Western blot or qPCR. **(j)** The effect of STAT3 knockdown on the viability of POLR2J-overexpressing cells was assessed by MTT assay. The results were presented as the Means ± standard deviation (SD), with n=3. *p < 0.05, **p < 0.01 vs. the control group; #*p* < 0.05, ##*p* < 0.01 vs. the POLR2J (KD) or POLR2J (OE).

To identify downstream targets of POLR2J, we integrated RNA-seq transcriptome data with bioinformatics analysis using UCSC and JASPAR databases. The analysis suggested GPX4 as a potential target gene of STAT3. Given the critical role of GPX4 in antioxidation and protecting cells from oxidative stress-induced damage, it was selected as a key candidate for further investigation. First, qRT-PCR and Western blot results showed that POLR2J knockdown significantly reduced GPX4 mRNA and protein levels in LUAD cells, whereas POLR2J overexpression markedly upregulated GPX4 expression (**Figures 6a, b**). Based on the mechanism by which RNA polymerase II initiates transcription through recruiting general transcription factors to form a pre-initiation complex, we hypothesized that POLR2J may indirectly regulate GPX4 expression through interaction with STAT3 (**Figure 6c**). Subsequent Co-IP experiments ruled out direct binding between POLR2J and GPX4; instead, they demonstrated that POLR2J overexpression enhanced its interaction with STAT3, while POLR2J knockdown attenuated this binding (**Figure 6d**).

To further determine whether STAT3 is a key factor directly regulating GPX4 expression, dual-luciferase reporter (**Figures 6e, f**) and ChIP-qPCR (**Figure 6g**) assays were performed. The results showed that STAT3 knockdown significantly reversed the increase in luciferase activity induced by POLR2J overexpression. Meanwhile, ChIP-qPCR assays confirmed that POLR2J promoted STAT3 binding to the GPX4 promoter region, and this enhancement was abrogated by STAT3 knockdown. Furthermore, qRT-PCR and Western blot analyses revealed that STAT3 knockdown reversed the POLR2J overexpression-induced upregulation of GPX4 at both mRNA and protein levels (**Figures 6h, i**). Additionally, cell viability assays demonstrated that STAT3 knockdown significantly suppressed the increased cell viability caused by POLR2J overexpression(**Figure 6j**). In conclusion, POLR2J knockdown induces cell death in LUAD cells by attenuating its interaction with STAT3 and subsequently inhibiting the STAT3/GPX4 signaling axis.

### 3.7 Overexpression of GPX4 reversed ROS-driven ferroptosis induced by POLR2J knockdown

**Figure 7:**
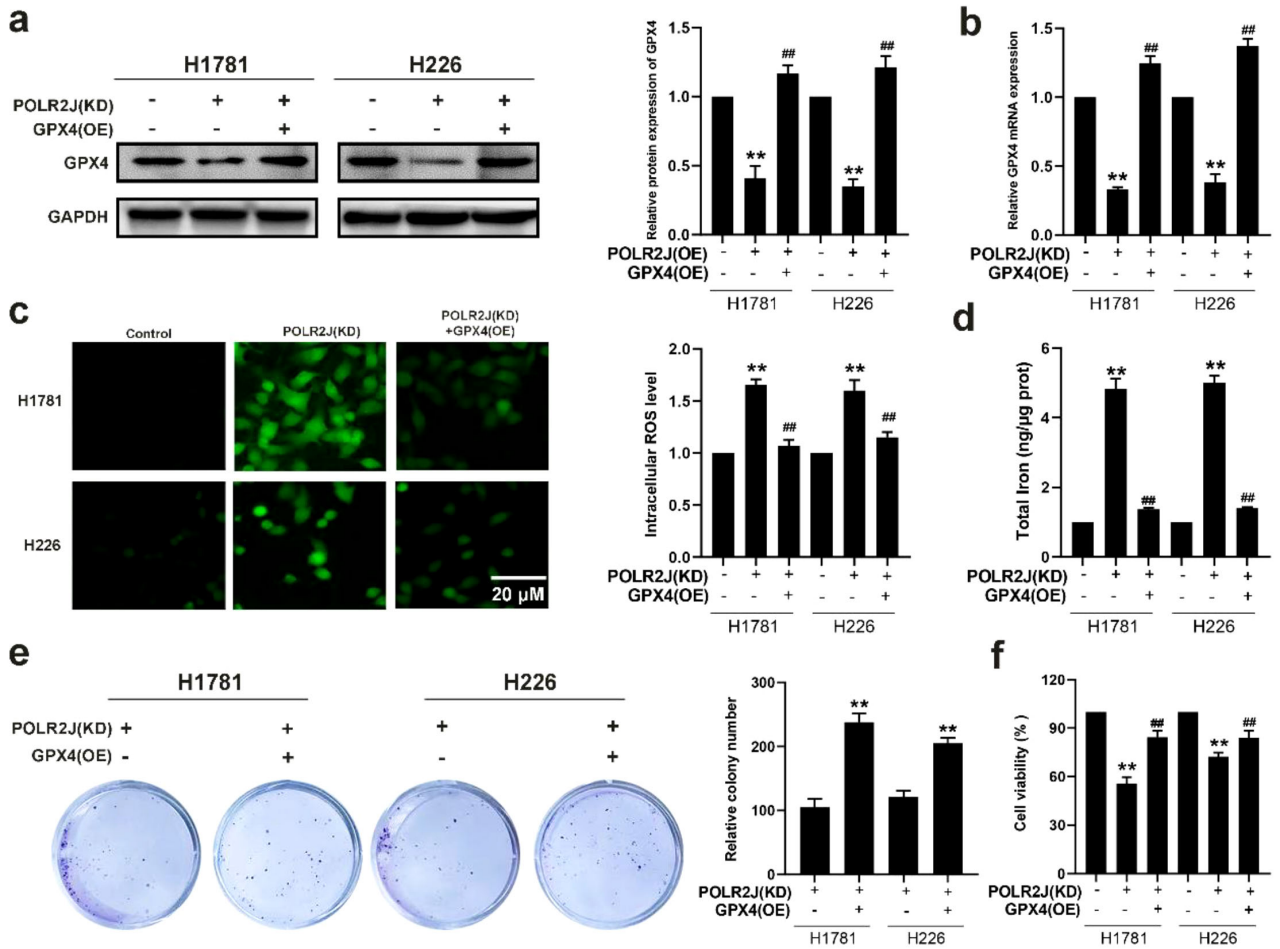
Overexpression of GPX4 reversed ROS-driven ferroptosis induced by POLR2J knockdown. **(a, b)** GPX4 overexpression was performed in POLR2J knockdown cells, and GPX4 expression levels were assessed by Western blot and qPCR. **(c)** Detection of reactive oxygen species (ROS) content in LUAD cell lines H1781 and H226 using DCFH-DA probe. **(d)** Effects of GPX4 overexpression on intracellular total iron levels in POLR2J knockdown cells. **(e)** Effects of GPX4 overexpression on the proliferative capacity in POLR2J knockdown cells as determined by clonogenic assay. **(f)** MTT assay of the effect of GPX4 overexpression on the proliferation in POLR2J knockdown cells. The results were presented as the Means ± standard deviation (SD), with n=3. *p < 0.05, **p < 0.01 vs. the control group; #*p* < 0.05, ##*p* < 0.01 vs. the POLR2J (KD) or POLR2J (OE).

To determine whether GPX4 mediates POLR2J knockdown-induced ferroptosis, rescue experiments were performed. As shown in **Figures 7a, b**, GPX4 overexpression restored GPX4 expression levels that were reduced by POLR2J knockdown at both protein and mRNA levels. Using the DCFH-DA probe, we found that the elevated ROS levels induced by POLR2J knockdown were significantly attenuated upon GPX4 overexpression (**Figure 7c**). Moreover, GPX4 overexpression reduced the intracellular total iron content in POLR2J knockdown cells (**Figure 7d**). Functionally, colony formation and MTT assays demonstrated that GPX4 overexpression rescued the proliferative capacity impaired by POLR2J knockdown (**Figures 7e, f**). Collectively, these results indicate that GPX4 overexpression reverses ROS-driven ferroptosis induced by POLR2J knockdown.

### 3.8 POLR2J promotes tumor growth in the nude mouse xenograft model

**Figure 8:**
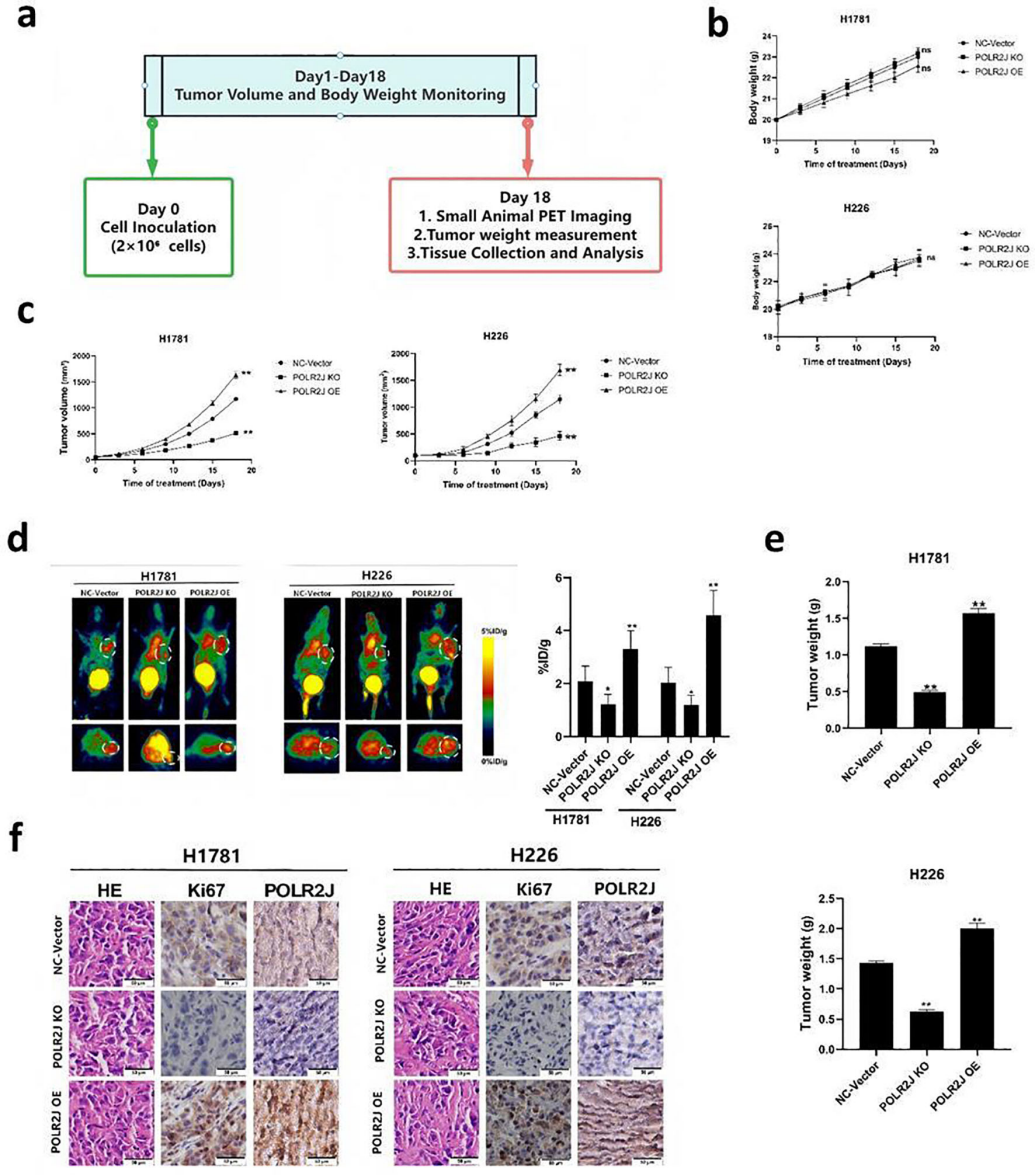
POLR2J regulates tumor growth in a nude mouse xenograft model. Based on the type of cells injected, the mice were assigned to three groups: NC-Vector, POLR2J knockdown (KO), and POLR2J overexpression (OE). **(a)** Schematic diagram of the animal experimental procedure. **(b, c)** Body weight curves and tumor growth curves of mice over the 18-day observation period. **(d)** Decay-corrected whole-body coronal microPET images of xenograft mice at 60 minutes after injection of 3.7 MBq of [18F]F-NOTA-PRGD2. The uptake of the quantitative tracer in the tumors and major organs is expressed as % ID/g. Tumors are indicated by white circles. **(e)** Final tumor weights at day 18. **(f)** Representative images of H&E staining, Ki-67 IHC, and POLR2J IHC in tumor tissues. The results were presented as the Means ± SD, with n = 5. **p < 0.01 *vs.* the control group.

To investigate the role of POLR2J in tumor growth *in vivo*, a nude mouse xenograft model was established (**Figure 8a**). Based on the type of cells injected, mice were assigned to three groups: NC-Vector, POLR2J knockdown (KO), and POLR2J overexpression (OE). During the 18-day observation period, no significant differences in body weight were observed among the three groups, whereas tumor growth curves showed that POLR2J KO significantly suppressed tumor growth and POLR2J OE markedly promoted tumor growth compared with the NC-Vector group (**Figures 8b, c**). Consistently, microPET imaging using [18F]F-NOTA-PRGD2 revealed that POLR2J KO decreased while POLR2J OE increased tracer uptake in tumors (**Figure 8d**). At the end of the experiment, final tumor weights were significantly lower in the KO group and higher in the OE group compared with the NC-Vector group (**Figure 8e**). Immunohistochemical analysis further demonstrated that POLR2J KO reduced Ki-67 positivity and POLR2J expression, whereas POLR2J OE showed the opposite effects (**Figure 8f**). These results indicate that POLR2J promotes tumor growth in the nude mouse xenograft model.

## 4 Discussion

The present study provides the first evidence linking POLR2J to LUAD patient survival and establishes POLR2J as an unexpected oncogenic driver in this disease. By demonstrating that POLR2J is markedly elevated in LUAD and tightly associated with advanced stage, lymph node metastasis and poor prognosis, and its expression is significantly higher in tumor tissues than in normal tissues regardless of gender, age, or smoking status, we position POLR2J as a previously unrecognized clinically relevant determinant of LUAD progression. Rather than functioning merely as a structural component of the transcriptional machinery, POLR2J emerges here as an active regulator of malignant behavior, influencing tumor cell proliferation, invasion, organoid growth, and migration.

Transcriptomic remodeling following POLR2J loss exposes its influence on multiple oncogenic circuits, including MAPK, PI3K-AKT, and JAK-STAT pathways, as well as biological processes tied to death and redox equilibrium. Consistent with these findings, evidence indicates that POLR2J interacts with STAT3 to promote the metastatic potential of glioblastoma cells (10, 16). STAT3 is a key transcription factor downstream of multiple cytokine and growth factor receptors, playing a particularly critical role in the JAK-STAT signaling pathway. Upon activation, STAT3 dimerizes and translocates to the nucleus, where it drives the transcription of genes involved in cell proliferation, survival, invasion, immune evasion, and the maintenance of stem-like properties (17). Persistent STAT3 activation is a hallmark of many malignancies and is closely associated with increased tumor aggressiveness and poor clinical outcomes (18). Together with our transcriptomic data, these observations suggest that POLR2J may modulate STAT3-dependent transcriptional programs and other oncogenic signaling cascades, thereby enhancing malignant traits and playing a central role in maintaining cellular homeostasis in LUAD.

A central finding of this study is the identification of POLR2J as a regulator of intracellular reactive oxygen species (ROS). Loss of POLR2J triggers ROS accumulation, DNA double-strand breaks and ferroptosis that are reversed by N-acetylcysteine (NAC), highlighting oxidative stress as a primary vulnerability unmasked by POLR2J depletion (19). ROS act both as cytotoxic oxidants and as critical signaling molecules, and their intracellular levels are tightly controlled to maintain cellular fitness. While basal ROS support proliferation through redox-sensitive pathways, excessive ROS drive oxidative damage, mitochondrial dysfunction and cell death (20). Cancer cells, including LUAD, typically operate near the threshold of oxidative toxicity owing to heightened metabolic activity and oncogenic signaling, rendering them heavily dependent on antioxidant systems to sustain redox homeostasis (21). Disruption of these buffering mechanisms therefore exposes a key metabolic fragility, whereby even modest increases in ROS can precipitate cell death. In line with this, our data show that POLR2J knockdown increases both ROS levels (rescued by NAC) and intracellular iron content, two key drivers of ferroptosis. Thus, POLR2J deficiency disrupts redox balance and sensitizes LUAD cells to iron-dependent cell death. Mechanistically, POLR2J sustains STAT3 phosphorylation, thereby engaging a transcriptional program that limits ROS-driven cytotoxicity. Given the well-established role of STAT3 in redox buffering and tumor cell survival (22), these data position POLR2J upstream of a canonical pro-tumorigenic signaling module (23). Functional rescue experiments further consolidate the POLR2J-STAT3 interaction: POLR2J overexpression mitigates the pro-oxidant effects of STAT3 silencing, and exogenous IL-6 reactivates STAT3 to suppress ROS under POLR2J loss. Together, these results delineate a POLR2J-STAT3-GPX4-ROS axis that endows LUAD cells with oxidative resilience and supports their survival.

**Figure 9:**
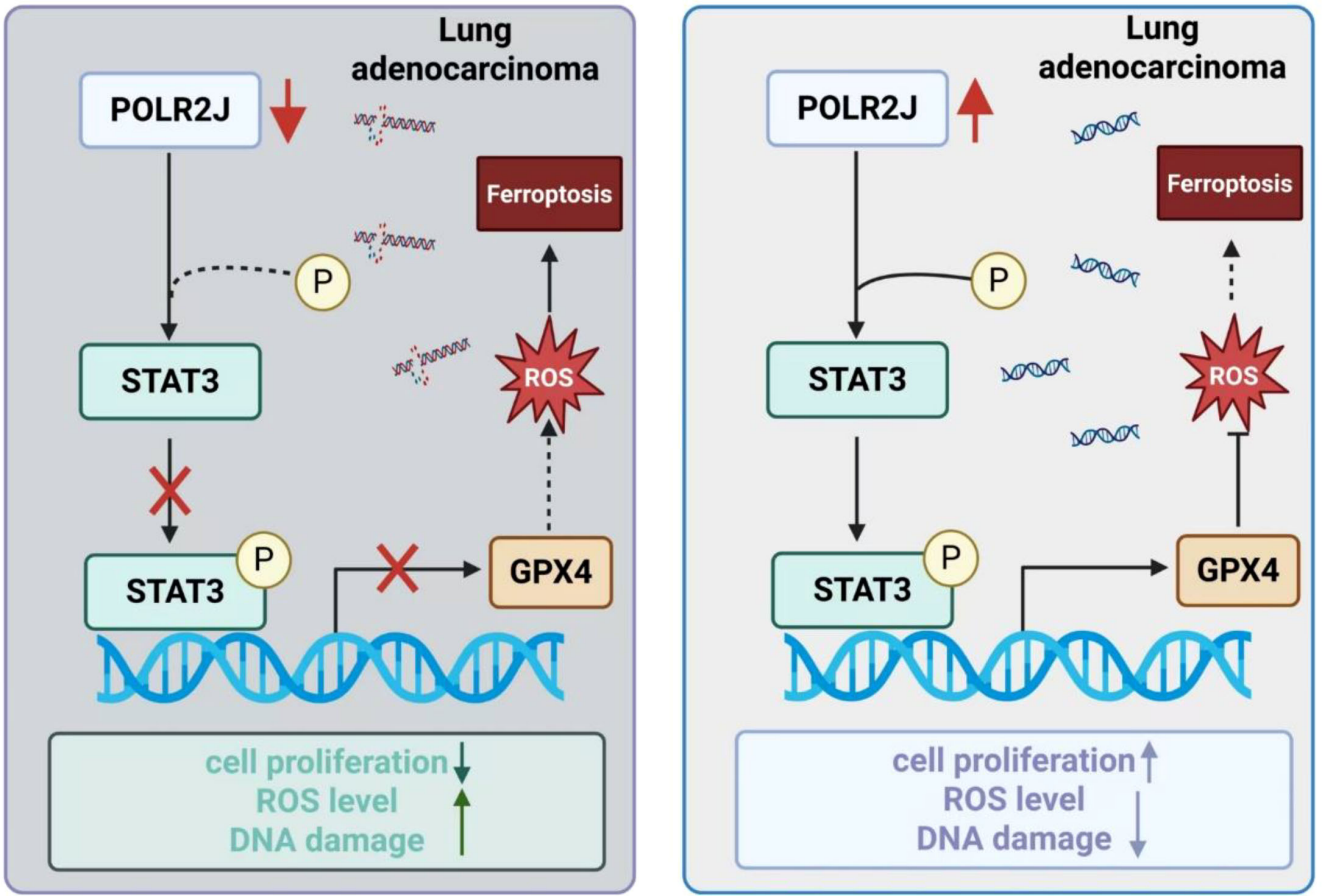
Schematic representation of the molecular regulatory mechanism of POLR2J in LUAD cells. (Generated via BioGDP.com).

By establishing POLR2J as a regulator of STAT3-GPX4-dependent redox control, this study fills a critical knowledge gap and identifies a previously unrecognized actionable node in LUAD biology. POLR2J thus emerges not only as a biomarker of aggressive disease but also as a molecular vulnerability that may be therapeutically exploited. Nevertheless, several limitations should be acknowledged: the upstream regulators driving POLR2J overexpression remain undefined; we did not include covariates such as gender, age, and smoking status in the multivariate analysis; and whether POLR2J confers sensitivity or resistance to existing LUAD therapies warrants further investigation. Moreover, During the analysis of transcriptomic data, we found that POLR2J knockdown led to transcriptional reprogramming, which may indicate impaired global transcriptional function caused by POLR2J deficiency. In summary, our study reveals that POLR2J upregulation drives LUAD proliferation and suppresses ROS-induced DNA damage via the STAT3-GPX4 axis, whereas its knockdown elicits opposing effects (**Figure 9**). However, the underlying mechanism requires further experimental investigation. We selected two representative LUAD cell lines (H1781 and H226) for experimentation. We acknowledge that the inclusion of additional lung adenocarcinoma cell lines in future studies would further strengthen the validity of our findings. Here, we demonstrate that POLR2J plays a critical role in transcriptional regulation and holds potential as a direct therapeutic target. However, we also recognize the challenges regarding specificity and potential toxicity that may accompany such targeting, which warrant further investigation. Addressing these questions will be essential for fully defining the clinical and therapeutic potential of targeting POLR2J in LUAD.

## 5 Conclusions

This study uncovers POLR2J as an important oncogenic determinant in the LUAD and delineates a previously unrecognized POLR2J-STAT3-GPX4-ROS signaling axis that shapes tumor growth, metastatic potential, and redox homeostasis. These findings redefine the functional landscape of POLR2J and highlight it as a promising therapeutic target, particularly for tumors reliant on STAT3-driven oxidative stress buffering. Targeting POLR2J or its downstream STAT3 circuitry may provide new avenues for intervention and holds potential for synergy with ROS-inducing anticancer strategies.

## Data Availability

The data presented in the study are deposited in the Mendeley Data repository (https://data.mendeley.com/), accession number DOI: 10.17632/82WD4CGY7.1.

## Supplementary material

Supplementary Figure 1 The ROS scavenger GSH can significantly reverse the DNA damage caused by POLR2J knockdown.

Supplementary Figure 2 The effects of POLR2J knockdown and overexpression on IL6 expression levels were detected by ELISA and RT-qPCR assays.
