# Supplementary figures and images for: POLR2J knockdown promotes ROS-induced DDR and ferroptosis by inhibiting the STAT3-GPX4 signaling axis in LUAD
Source: Front Oncol. 2026 Apr 15;16:1787943. doi: 10.3389/fonc.2026.1787943 (PMC13124589; doi:10.3389/fonc.2026.1787943)

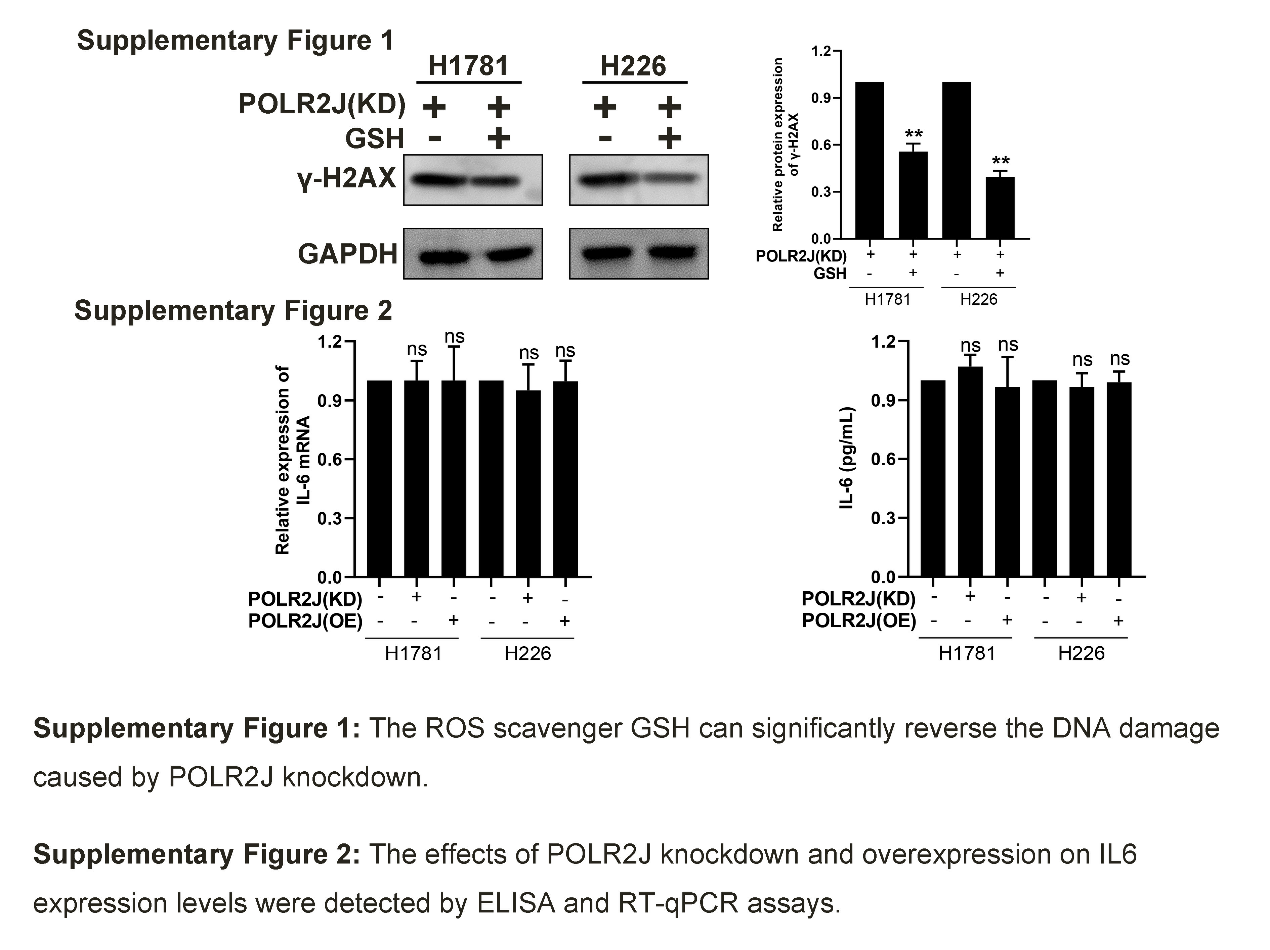

Supplement: Supplementary Figure 1 — The ROS scavenger GSH can significantly reverse the DNA damage caused by POLR2J knockdown. [file DataSheet1.docx]
